# Highly functional T-cell receptor repertoires are abundant in stem memory T cells and highly shared among individuals

**DOI:** 10.1038/s41598-017-03855-x

**Published:** 2017-06-16

**Authors:** Takahiko Miyama, Takakazu Kawase, Kazutaka Kitaura, Ren Chishaki, Masashi Shibata, Kumi Oshima, Hiroshi Hamana, Hiroyuki Kishi, Atsushi Muraguchi, Kiyotaka Kuzushima, Hiroh Saji, Tadasu Shin-I, Ryuji Suzuki, Tatsuo Ichinohe

**Affiliations:** 1 Department of Hematology and Oncology, Research Institute for Radiation Biology and Medicine (RIRBM), Hiroshima University, Hiroshima, Japan; 2 Depatment of Rheumatology and Clinical Immunology, Clinical Research Center for Allergy and Immunology, Sagamihara National Hospital, National Hospital Organization, Sagamihara, Japan; 3 Department of Immunology, Graduate School of Medicine and Pharmaceutical Sciences, University of Toyama, Toyama, Japan; 4 Division of Immunology, Aichi Cancer Center Research Institute (ACC), Nagoya, Japan; 5 HLA Foundation Laboratory, Kyoto, Japan; 6 BITS Incorporation, Tokyo, Japan

## Abstract

To expand our knowledge of the ontogeny of the T-cell receptor (TCR) repertoire of antigen-specific T-cell subsets, we combined next-generation deep sequencing and single-cell multiplex clonotype analysis to evaluate the diversity and frequency of paired TCRs, their functions and whether clonotypic TCRs are shared among different individuals. Using an HLA-A*02-restricted cytomegalovirus (CMV) pp65-derived immunogenic peptide, we found that the more dominant pp65-specific TCR clonotypes in the blood of healthy donors have higher binding affinities for the CMV peptide and arise from clonotypes that are highly shared among individuals. Interestingly, these highly shared HLA-A*02-restricted CMV-specific TCRs were detected in a CMV-seronegative individual as well as in HLA-A*02-negative donors albeit at lower frequency. More intriguingly, these shared TCR clonotypes were abundant in the stem memory T-cell subset, and TCR diversity of the stem memory T-cell repertoire was significantly lower than in the central memory and effector memory T-cell repertoires. These results suggest that the stem memory T-cell subset may serve as a reservoir of highly shared and highly functional memory T-cells.

## Introduction

High-throughput next-generation sequencing (NGS) of rearranged T-cell receptor (TCR) gene segments comprising the variable (V), diversity (D; for the TCR beta- and delta-chains), and joining (J) regions is being increasingly used to conduct comprehensive simultaneous analyses of human T cell populations in healthy and diseased individuals^1, 2^. The vast amount of information acquired through NGS analysis of T-cell receptor (TCR) clonotypes greatly surpasses that using conventional methods such as flow cytometry or spectratyping. NGS facilitates deep analysis of the T-cell clones in a manner sufficient to obtain a landscape of the TCR repertoire in a given sample or to trace very rare T cell populations that were not previously identifiable. Although NGS is a powerful tool for elucidating the T-cell repertoire at high resolution, a caveat is that this technology analyses the TCR alpha (α) (*TRA*) and TCR beta (β) (*TRB*) loci separately. Therefore, exact high-throughput pairing of the identified TCRα and TCRβ subunits is usually difficult. However, without this information, it is difficult to evaluate the antigen specificity of the identified TCRs and to conduct functional assays using T cells engineered to express those paired TCRαβ subunits. To mitigate this disadvantage, we have developed an integrated methodology that comprehensively and simultaneously analyses paired TCRαβ repertoires and their function by combining semi-quantitative NGS with single-cell multiplex clonotypic analysis to clarify pairs of TCRαβ expressed on T cells at a single-cell level (designated as human T cell efficient cloning within 10 days, hTEC10)^3^.

Since the existence of a stem-cell memory (SCM) T-cell subset was first proposed^4, 5^, immunological and molecular genetic studies have identified the SCM T-cell subset in mice, non-human primates and humans. Although the clinical importance of the SCM T-cell subset has been suggested, particularly as a source for adoptive gene-edited or -transduced T-cell therapy, the mechanisms by which the TCR repertoire of the SCM T-cell subset is developed from a huge number of circulating TCR clonotypes remain unexplored. Further, a differentiation hierarchy of functional T-cell subsets (naïve T cells, SCM T cells, central memory [CM] T cells, effector memory [EM] T cells and terminal effector [EFF] T cells) of the human immune system remains to be defined in more detail. For example, evidence indicates that SCM T cells are less differentiated than CM and EM T cells and that the path of differentiation of T cells is unidirectional^6^. However, other models have proposed that effector cells are a source for EM T cells that subsequently give rise to CM T cells, or hypothesized the existence of a divergent generation of different memory T-cell subsets.

The TCR repertoire is generated through TCR gene recombination, thymic selection and peripheral immunological modulation by foreign antigens. TCRs generated by these three steps are screened to eliminate detrimental variants. Previous studies have revealed more frequent expression of certain TCR sequences through V-(D)-J gene recombination compared with others by the mechanism of convergent recombination and recombinatorial enzyme bias. Convergent recombination is the process whereby multiple recombination events converge to produce the same nucleotide sequence encoding the same CDR3 amino-acid sequence^7–10^. Whereas, enzyme bias is another mechanism where some enzymes confer biased recombination of TCR genes leading to production of T cells harboring identical TCR amino acid sequences among different individuals^11–13^. Moreover, certain well-screened functional TCRs shared between HLA-matched individuals are designated as public or shared TCRs^7^.

The primary objective of the present study was to semi-quantitatively determine the TCR repertoire of functional T-cell subsets and antigen-specific T cells with emphasis on the diversity among T-cell subsets and to estimate the frequency of “shared” TCR clonotypes among different individuals. We focused on T cells that recognize a highly immunogenic HLA-A*02-restricted peptide derived from cytomegalovirus (CMV) pp65, which served as a model of virus-specific CD8+ T-cell responses. The secondary objective was to determine the antigen specificity and function of specific TCRs and then to clarify the association between the sharing of TCRs among individuals and the functions of these shared TCRs.

Our results indicate that highly functional CMV-specific TCR clonotypes are abundant in each functional T-cell subset and are frequently shared among individuals. Further, the more dominant CMV pp65-specific clonotypes have higher affinity and arise from more highly shared clonotypes. Interestingly, these highly shared HLA-A*02-restricted CMV-specific TCRs were detected in a CMV-seronegative donor and HLA-A*02-negative donors. We further showed that the TCR repertoire of SCM T cells is less diverse than those of the CM and EM T-cell subsets, suggesting that the SCM T-cell subsets serve as reservoirs of highly functional memory T cells that express TCRs highly shared among individuals. The integrated high-throughput technology used here to evaluate paired TCRαβ repertoires will confer new insights into the structure and function of TCRs responsible for a variety of human immune responses. These findings may lead to the future development of individualized TCR gene-transduced adoptive immunotherapy.

## Results

### Diversity of the unfractionated and CMV pp65-NLV-specific T-cell repertoires among five healthy donors

We first used NGS and single-cell multiplex clonotypic analysis to analyse the entire and CMV NLV-specific T-cell repertoires in the blood of five healthy donors whose characteristics are shown in Table 1. All donors were screened for CMV serostatus and typed for HLA class I and II alleles. Three donors were HLA-A*02:01-positive (V001) or HLA-A*02:06-positive (V002 and V004), and four donors were CMV seropositive (V001, V002, V004 and V005).

**Table 1:** Donor characteristics.

Donor	Age, y	Sex	CMV serostatus	HLA-A typing
V001	50	Male	+	*02:01/*24:02
V002	41	Male	+	*02:06/*24:02
V003	22	Male	−	*11:01/*24:02
V004	23	Male	+	*02:06/*24:02
V005	36	Male	+	*26:03/*24:02

**Figure 1:**
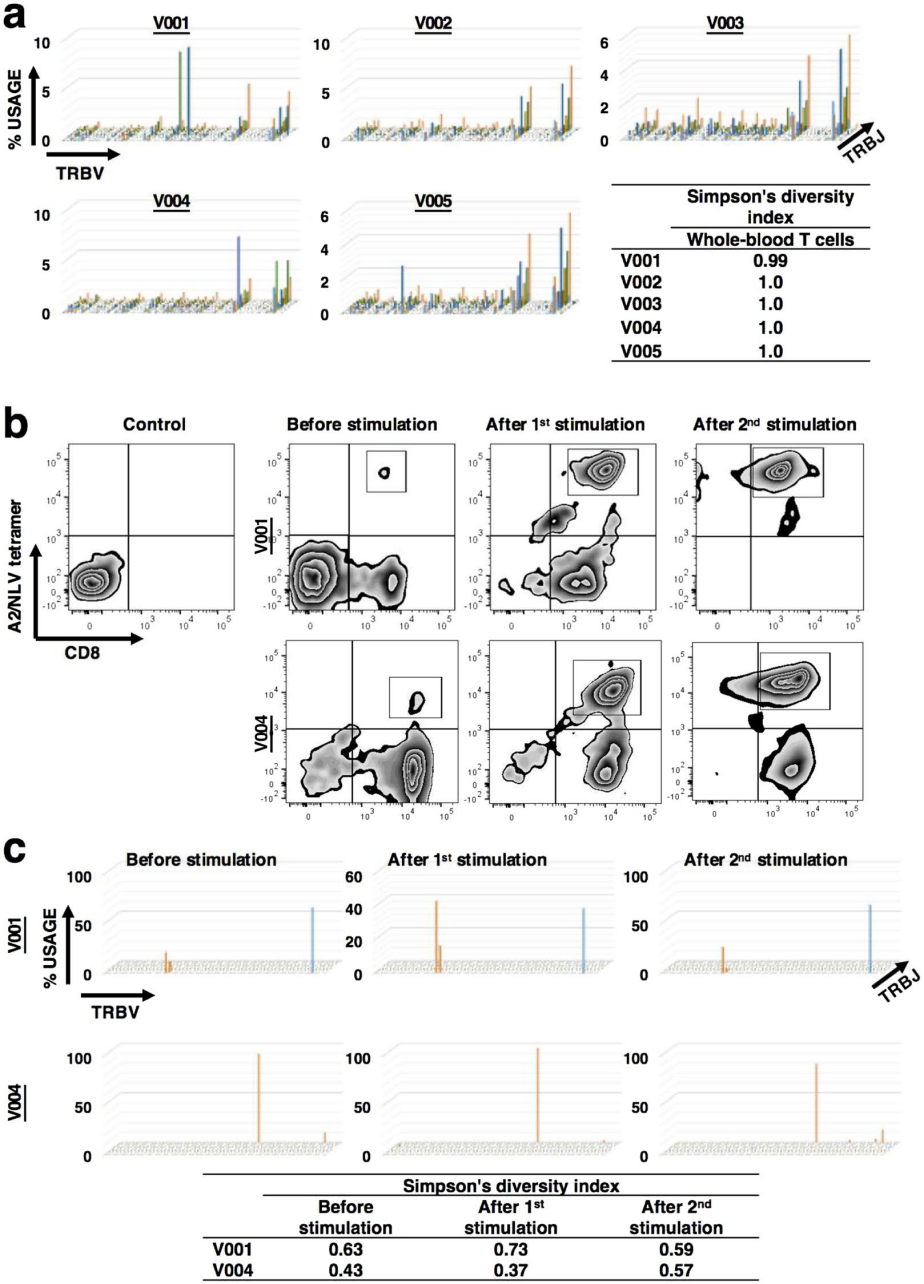
Diversities of the entire T cell repertoires and CMV NLV-specific T cell repertoires among five healthy donors. (**a**) NGS of the T-cell repertoire identifies specific CDR3 amino acid sequences and expression of TCRβ variable (*TRBV*)- and joining (*TRBJ*)-region genes. Diversity Index and TCR repertoire plots illustrate the wide range of diversity of the total T cell populations of five healthy donors (V001, V002, V003, V004 and V005). (**b**) Representative flow cytometric profiles of unstimulated and stimulated PBMCs derived from V001 (HLA-A2-positive and CMV-seropositive healthy donor) for HLA-A2-NLV tetramer-positive cells. PBMCs were stimulated once or twice with autologous antigen-presenting cells pulsed with the peptide NLVPMVATV (NLV) *in vitro*. The stimulation protocol is detailed in the Methods section. (**c**) T-cell repertoire plots and Diversity Index of CMV NLV-specific CD8+ T cells sorted from unstimulated and stimulated PBMCs from two healthy donors (V001 and V004; HLA-A2-positive and CMV-seropositive healthy donors, respectively). CMV NLV-specific CD8+ T cell repertories are oligoclonal before and after antigen stimulation.

We conducted NGS analysis of equivalent numbers of PBMCs obtained from the five donors (Table 1). Grouping *TRBV* and *TRBJ* expression and counting the number of reads within each group illustrates the diversity of the entire T-cell repertoire (Fig. 1a). We evaluated the diversity of the unfractionated entire T-cell repertoire among the donors by calculating Simpson’s Diversity Index (SDI) using the NGS data. The indexes ranged from 0.99 to 1.00 (average, 1.00) (Fig. 1a), which indicates the high diversity of their entire T-cell populations.

Next, we investigated the diversity of the virus-specific T-cell repertoires among these donors. NLV-peptide is an HLA-A*02-restricted epitope derived from CMV pp65 matrix protein and serves as a model antigen of virus-specific CD8+ T cell responses. NLV is highly immunogenic in individuals expressing HLA-A*02^8, 9^. The HLA-A*02-NLV tetramer was used to detect CMV NLV-specific CD8+ T cells. Flow cytometric analysis of unstimulated PBMCs was performed, and HLA-A*02-NLV tetramer-positive cells were detected in samples from V001 and V004 (Fig. 1b). Next, CD8+ T cells derived from the five donors were stimulated with the NLV peptide once each week for two weeks. HLA-A*02-NLV tetramer-positive cells were detected only in the NLV peptide-stimulated cells from V001 and V004 after one and two weeks (Fig. 1b), even after two rounds of treatment with the NLV peptide.

The results of NGS analysis revealed that CMV NLV-specific CD8+ T-cell repertories in the peripheral circulation were oligoclonal irrespective of the presence or absence of antigenic stimulation (Fig. 1c). Respective SDIs of CMV NLV-specific CD8+ T cell repertories of V001 and V004 were 0.63 and 0.43 in unstimulated samples, 0.73 and 0.37 after one round of stimulation, and 0.59 and 0.57 after two rounds of stimulation, which indicates the oligoclonality of CMV NLV-specific CD8+ T cell repertories. Random sampling was performed to confirm that this result was not affected by the difference in the read number of each sample. As a result, the differences between SDIs calculated using all reads and average of those calculated using random sampling data were less than 0.05% (Supplementary Table 1).

### Characterization of CMV NLV-specific TCRα and TCRβ repertoires of single cells

**Figure 2:**
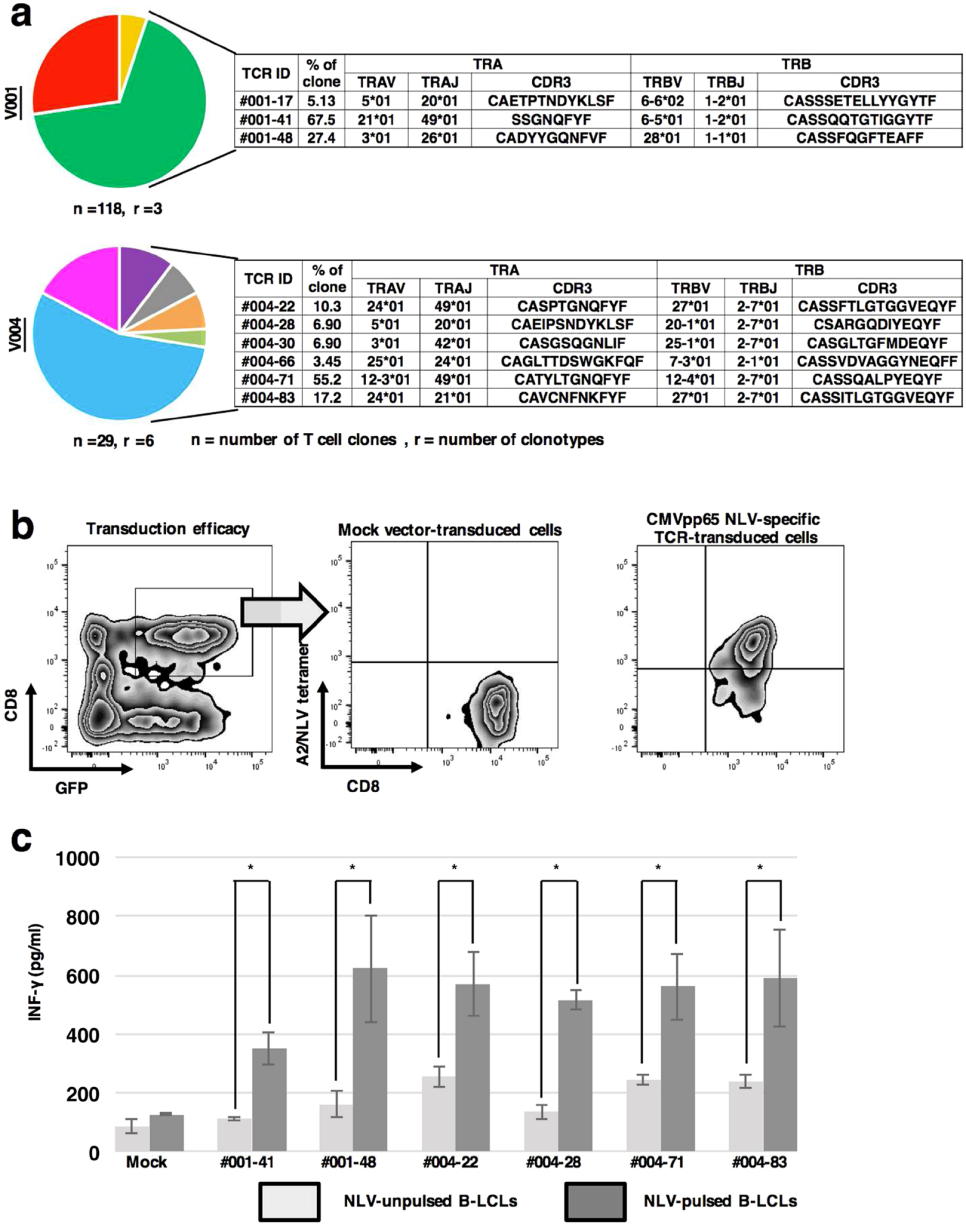
Characterization of CMV NLV-specific TCRα and TCRβ repertoires of single cells. (**a**) CMV NLV-specific TCRα and TCRβ repertoires identified using single-cell multiplex clonotypic analysis of two HLA-A2-positive and CMV-seropositive healthy donors (V001 and V004). Single CMV NLV-specific T cells were sorted into 96-well PCR plates and then cDNAs were amplified using multiplex RT-PCR. The PCR products were sequenced and conceptually translated to identify CDR3α and CDR3β. We analysed 118 and 29T cells from V001 and V004, respectively, and identified 3 (TCR ID; 001-17, 48 and 41) and 6 (TCR ID; 004-66, 22, 63, 30, 28 and 71) CDR3α and CDR3β pairs from each respective donor. (**b**) Transduction of sequences encoding CMV NLV-specific TCRs. TCRα and TCRβ pairs were cloned into expression vectors encoding GFP that were used to transfect PHA blasts derived from a CMV-seronegative healthy donor. Representative flow cytometric profiles illustrate transduction efficiencies. Cells with double-positive staining for the NLV tetramer and GFP were considered successful transductants. (**c**) IFN-γ production by TCR-transduced cells. TCR-transduced cells were co-cultured for 16 h with NLV-pulsed and NLV-unpulsed B-LCLs derived from the cognate donors. The IFN-γ concentrations in culture supernatants were measured using an IFN-γ ELISA. The results represent the mean ± standard deviation (S.D.) of triplicate experiments. The error bars represent standard deviation. The asterisks indicate significant difference (p < 0.05) in the IFN-γ concentrations between samples co-cultured with NLV-pulsed and NLV-unpulsed B-LCLs (paired t-test). Although mock-transduced PHA blasts did not recognize NLV peptide-pulsed or control B-LCLs, all TCR-transduced PHA blasts recognized only NLV peptide-pulsed B-LCLs, verifying the antigenic specificity of the TCR-transduced PHA blasts.

Concurrently with NGS analysis, we used single-cell multiplex clonotypic analysis (hTEC10) of the CDR3 regions of *TCRA* and *TCRB* gene segments to identify CMV NLV-specific TCRα and TCRβ repertoires. For this purpose, these paired TCR gene segments identified in a single CMV NLV-specific T cell were used to transduce PHA blasts derived from CMV seronegative donors. Single CMV NLV-specific T cells sorted from unstimulated PBMCs derived from V001 and V004 were used to generate cDNAs that were subjected to Sanger sequencing to identify sequences encoding the CDR3α and CDR3β domains. We determined TCR sequences of 29 and 118 CMV NLV-specific T cells from V001 and V004, respectively, and found that there were three (TCR IDs 001–17, 48 and 41) and six (TCR IDs 004–66, 22, 63, 30, 28 and 71) TCRαβ-paired clonotypes in the samples acquired from donors V001 and V004, respectively (Fig. 2a). These results revealed that CMV NLV-specific T-cell repertories harbored a few unique dominant clones and other less dominant clones.

To characterize the functions of the CMV NLV-specific TCRs identified using single-cell multiplex clonotypic analysis, these TCRs were cloned into a GFP-expression vector, which was used to transfect the TCRs into PHA blasts derived from the CMV-seronegative healthy donor. Transduction efficacy was verified using flow cytometry with the HLA-A*02-NLV tetramer as the probe. The detection of double-positive cells (HLA-A*02-NLV tetramer/GFP) is shown in Fig. 2b.

To confirm antigenic specificity of TCR-transduced PHA blasts, cells were co-cultured for 16 h with NLV-pulsed and untreated B-LCLs derived from the respective cognate donors of the PHA blasts. IFN-γ concentrations in the culture supernatant were measured using an ELISA. Although mock-transduced PHA blasts did not recognize NLV peptide-pulsed or untreated B-LCLs, all TCR-transduced PHA blasts reacted with NLV peptide-pulsed B-LCLs to produce IFN-γ but not with NLV-unpulsed B-LCLs (Fig. 2c), verifying the antigenic specificity of the TCRs to the NLV/HLA-A2 complex.

### Binding affinities of dominant and subdominant CMV NLV-specific TCRs

**Figure 3:**
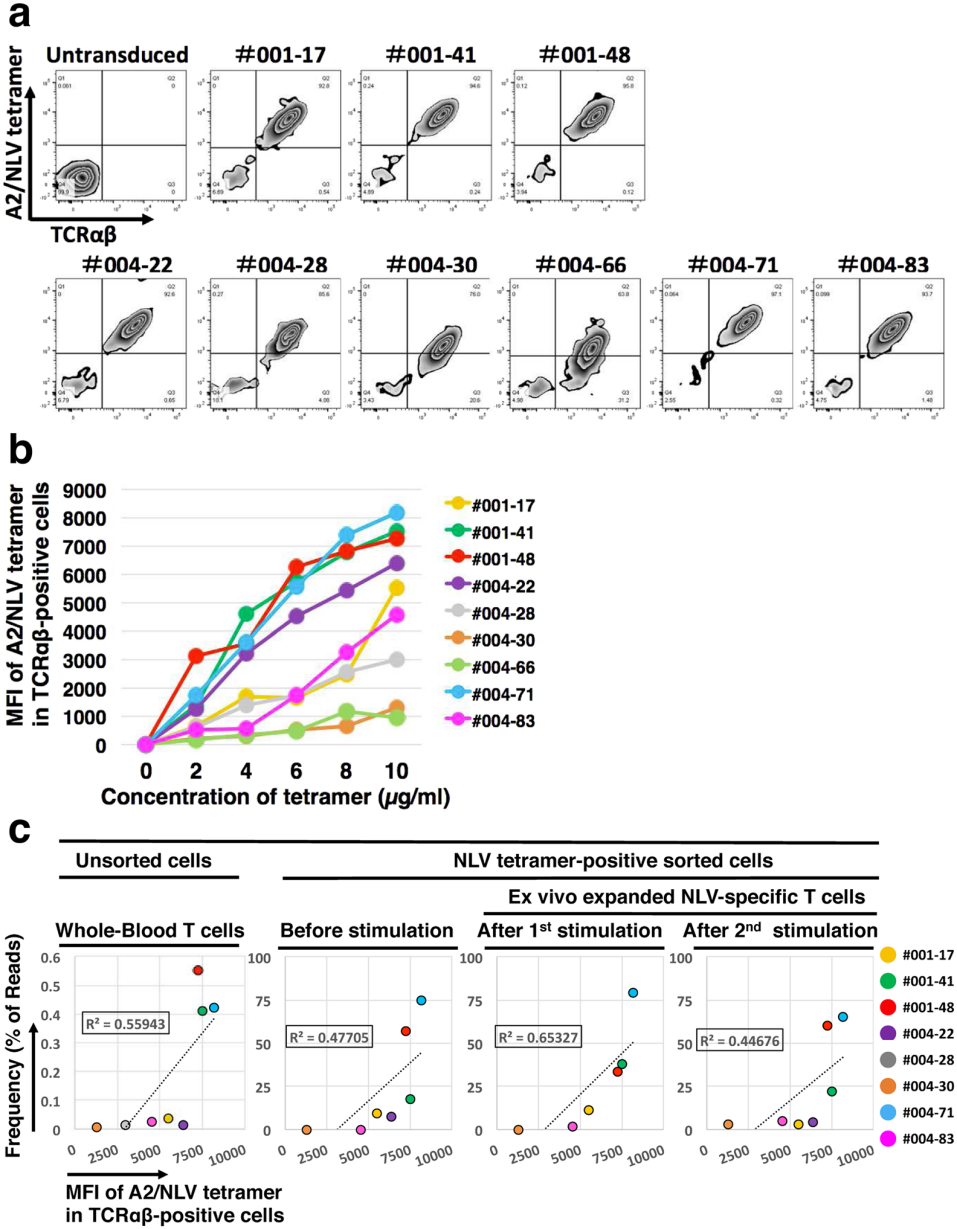
Binding affinities of the dominant and subdominant CMV NLV-specific TCRs. (**a**) Flow cytometry was used to measure the levels of TCR expression by TCR-transductants and to determine the binding affinities of TCRs for the NLV peptide/MHC complex. Sequences representing the TCRs identified using single-cell multiplex clonotypic analysis were used to transfect Jurkat cells that lack TCRαβ expression that were probed using the HLA-A2-NLV tetramer-PE and anti-TCRαβ-APC. (**b**) Binding analysis of the dominant and subdominant CMV NLV-specific TCRs. Jurkat cells that lack TCRαβ expression transduced with CMV NLV-specific TCRs were reacted with HLA-A2-NLV tetramer-PE at the indicated concentrations. Mean fluorescence intensity (MFI) of the cells is plotted vs HLA-A2-NLV-tetramer concentration. (**c**) The relationship between the MFIs of Jurkat cells that lack TCRαβ expression transduced with CMV NLV-specific TCRs and the frequencies of clonotypes were estimated according to the percentage of the NGS reads of the TCR sequences. MFI values of Jurkat cells that lack TCRαβ expression transduced with CMV NLV-specific TCRs are plotted vs the estimated frequency of the T-cell clonotypes (left panel) of sorted cells from unstimulated (second left panel), one (second right panel) and two rounds (right panel) of stimulated T cells. Correlation was assessed using Spearman’s rank correlation coefficient. All samples in this analysis were derived from CMV-seropositive and HLA-A2-positive donors, V001 and V004, respectively. The frequencies of the CMV NLV-specific T-cell clones in samples were estimated from the number of unique reads that correlated positively with the binding affinities of the TCRs. The correlation coefficients are shown in each plot. The correlation was consistently independent of the number of antigen stimulations.

To determine the binding properties of CMV NLV-specific TCRs, we established Jurkat cells genetically engineered to lack endogenous TCR expression by CRISPR-Cas9 system. These TCR-null Jurkat cells were transduced with CMV NLV-specific TCRs and were tested for their binding to the HLA-A*02-NLV tetramer (Fig. 3a). The TCR-null Jurkat cells transduced with TCR001-41, 001-48 and 004-71, which were the most dominant CMV NLV-specific TCR clonotypes of V001 and V004, were capable of binding to the A*02/NLV complex with significantly higher affinities compared with the others (*P* = 0.05) (Fig. 3b). The estimated frequencies of these three dominant TCRs among all CMV NLV-specific T-cell clonotypes were 67.6%, 27% and 55.1%, respectively (Fig. 2a). The frequencies of CMV NLV-specific T-cell clones in samples estimated according to the number of total reads correlated positively with binding affinity. The correlation was consistent irrespective of the presence or absence of one or two rounds of antigenic stimulation (Fig. 3c).

### Analysis of shared TCRs and functional CD8+ T-cell subsets of five healthy donors

Immunodominant CMV-specific TCR clonotypes (“public” TCRs) are frequently shared among HLA-matched individuals^7^. Therefore, we further aimed to investigate the sharing of TCR repertoires among the five donors. We focused on CMV NLV-specific TCRs as well as their precursors expressed by naïve and functional memory CD8+ T-cell subsets. We defined shared TCRs as TCR clonotypes encoded by identical *TRBV/TRBJ* genes and CDR3 amino acid sequences expressed by at least two donors. According to this definition, the estimated frequencies of shared and unshared TCRs were calculated as the proportion of the read number of relevant unique reads versus the total number of informative reads of the cDNAs prepared from whole-blood T cells.

**Figure 4:**
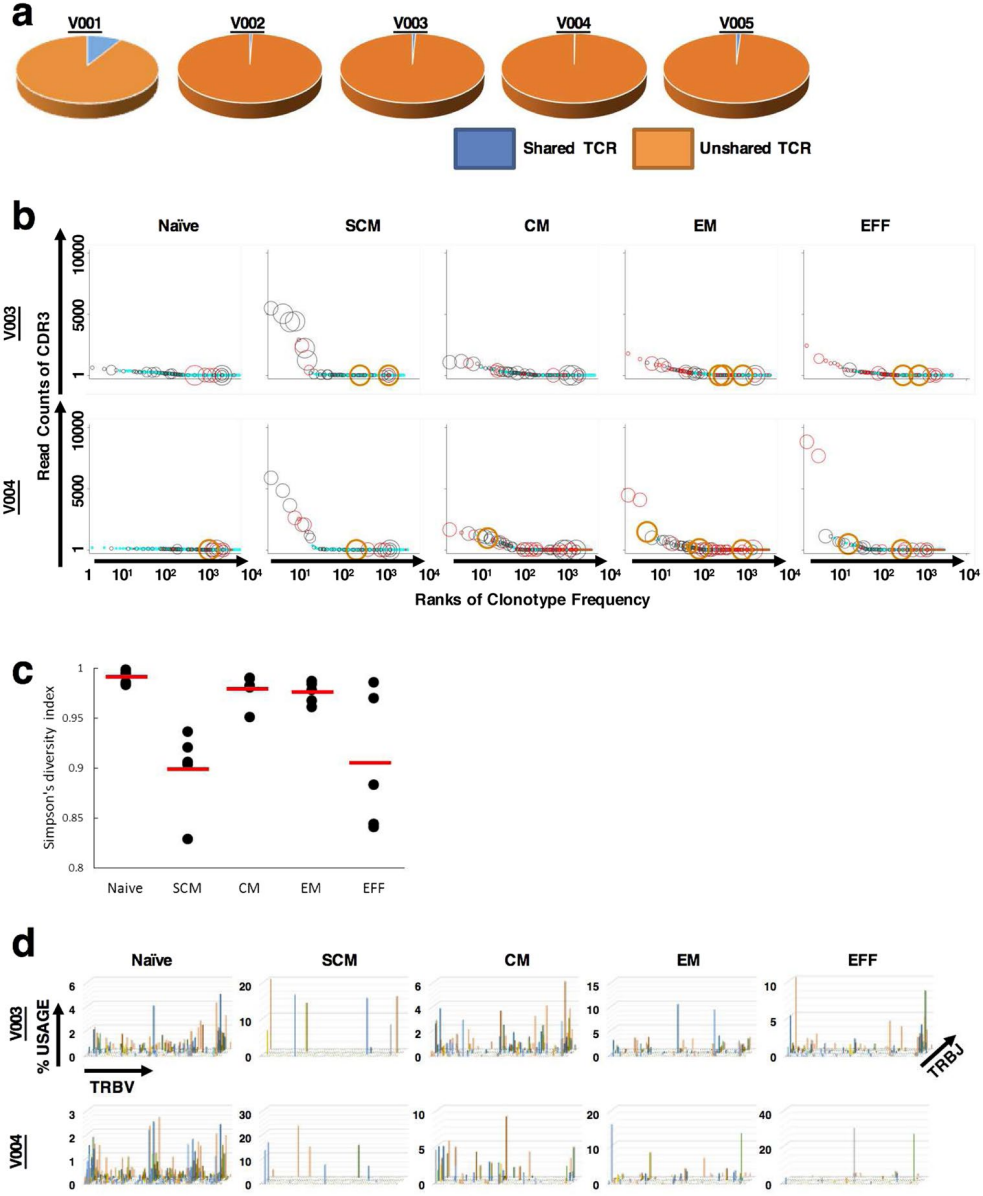
Shared TCRs among five healthy donors present in their populations of T cells and functional CD8+ T cell subsets. (**a**) Ratio of unique reads of shared and unshared TCRs in the entire T-cell populations. The pie charts represent the total number of unique reads of TCRs in the entire T cell populations. Blue and orange indicate the percentage of unique reads of shared and unshared TCRs, respectively. (**b**) Random sampling of 35,000 reads was performed in each T cell subset and each donor. Each circle or dot in the plots shows one unique read of TCRβ. TCRβ clonotypes are ordered according to their frequencies in descending order on the x-axis. The y-axis depicts the read numbers of the clonotypes. Shared non-CMV NLV-specific TCRs (○) and unshared non CMV NLV-specific TCRs (●) are shown as black circles and blue dots, respectively. Shared CMV NLV-specific TCRs (○) and unshared CMV NLV-specific TCRs (●) are shown as red circles and dots, respectively. Orange thick circle (○) represents one of nine dominant TCRs in V001 (TCR ID; 001-17, 001-48, 001-41) and V004 (TCR ID; 004-66, 004-22, 004-63, 004-30, 004-28 and 004-71) (Fig. 2a). The sizes of the circles are directory proportional to numbers shared among the five donors. The largest circles indicate the shared clonotypes by the five donors and the dots indicat the unshared clonotypes. Wilcoxon rank-sum test and Kendall’s tau test were performed. The P values of all tests were <0.05, which indicates a significant correlation between the total reads and the sharing count of the TCRs, even in the naïve subset. The results of this analysis were similar for donors V002, V003 and V005 (Supplementary Fig. 2). (**c**) Simpson’s Diversity Index (SDI) calculated using NGS data for functional T-cell subsets among the five donors. The y-axis depicts SDI, and each dot represents each donor’s SDI of functional T-cell subsets. The horizontal bars show the average SDI among the five donors. (**d**) T cell repertoire plots of functional CD8+ T-cell subsets are the same as shown in Fig. 4c, revealing the lower diversity of the SCM T-cell subset compared with CM and EM T-cell subsets. The results of this analysis were similar for donors V002, V003 and V005 (Supplementary Fig. 3).

The estimated frequencies of shared TCRs among the five donors ranged from 0.60% to 8.67% (average 2.26%) (Fig. 4a). We further investigated the frequencies of shared and unshared TCR clonotypes in naïve, SCM, CM, EM, and EFF CD8+ T-cell subsets, which were subjected to NGS analysis. The frequencies of unique and total reads of shared and unshared TCRβ-chains are summarized in Supplementary Table 2. Although the percentage of unique reads of shared TCRs was very small, shared TCRs were more frequently present compared with unshared TCRs in every functional T-cell subset.

To investigate the association between sharing of TCR clonotypes among individuals and the frequency of TCR clonotypes, each TCR clonotype was plotted according to the NGS read number of functional T-cell subsets shared by the five donors (Fig. 4b and Supplementary Fig. 1). Shared TCRs were more abundantly present compared with unshared TCRs, and the read number of shared TCRs tended to be higher, even in the naïve subset irrespective of the donor (Fig. 4b and Supplementary Fig. 1).

To investigate the change of diversity during T-cell differentiation, SDIs of each T-cell subset were calculated (Fig. 4c). As we expected, naïve T-cell subsets were the most diverse (average 0.99, range 0.98–1.00), followed by the CM T-cell (average 0.98, range 0.95–0.99), and EM T-cell subsets (average 0.97, range 0.96–0.99). Interestingly, the SCM T-cell subset was less diverse (average 0.90, range 0.82–0.94) than the CM and EM T-cell subsets. Further, SDI of the SCM T-cell subset was similar to the index of the EFF T-cell subset (average 0.91, range 0.84–0.97), which is consistent with the *TRBV* and *TRBJ* data (Fig. 4d and Supplementary Fig. 2). To confirm that this result was not affected by different read number of each sample, we also performed random sampling and calculated SDI for all 25 samples^10^. The number of random sampling was 35,000 each, which were the round number less than the size of the smallest sample (the read number of SCM sample of V005; 39667). The number of repetitions was 100 each. As a result, the differences between SDIs calculated using all reads and average of those calculated using random sampling data were less than 0.02% in all 25 samples (Supplementary Table 1). These observations suggest that SCM T-cell subsets serve as a reservoir of highly shared and highly functional memory T cells present at relatively high frequency.

### Shared and unshared TCRs of the CMV NLV T-cell repertoire of two CMV-seropositive donors

**Figure 5:**
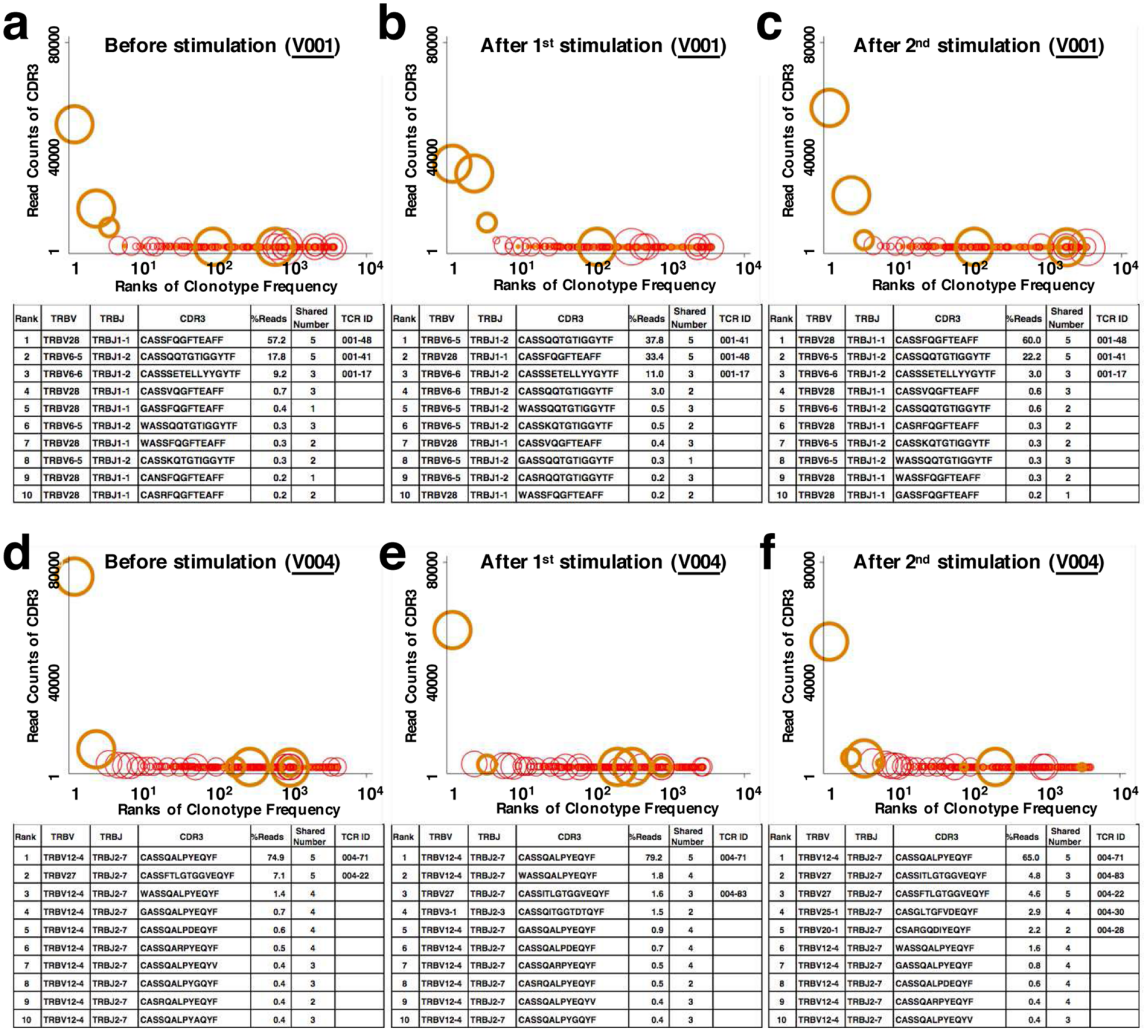
Distributions of read numbers of CMV-NLV-specific TCRβ clonotypes. Cells from the HLA-A2-positive and CMV-seropositive donors V001 and V004 that were detected using the HLA-A2-NLV tetramer probe were sorted and isolated from unstimulated PBMCs (**a**,**d**) and PBMCs stimulated once (**b,e**) or twice (**c,f**) with the NLV peptide. The sorted cells were then subjected to NGS. Each marker in the plots shows one unique read of CMV NLV-specific TCRβ. The TCRβ clonotypes are ordered according to their frequencies on the x-axis. The y-axis depicts the read number of the clonotypes. Shared CMV NLV-specific TCRs and unshared CMV NLV-specific TCRs are shown as red circles and red dots, respectively. Orange thick circle represents one of nine dominant TCRs in V001 (TCR ID; 001-17, 001-48, 001-41) and V004 (TCR ID; 004-66, 004-22, 004-63, 004-30, 004-28 and 004-71) (Fig. 2a). The sizes of the circles represent the number of sharing (two to five) among the five donors. The largest circle indicates that the corresponding TCR clonotype were shared by the five donors. The Wilcoxon rank-sum test was performed to assess whether public CMV NLV TCRs occupy a higher rank of the read numbers compared with the private CMV NLV TCRs. Wilcoxon rank-sum test and Kendall’s tau test were performed. All tests were statistically significant (P < 0.05). The ranks (the first to tenth) of %read of CMV NLV-specific TCRβ clonotypes are shown in the tables below the plots.

The numbers of unique *TRB* reads of the CMV NLV T-cells in unstimulated PBMC samples from donors V001 and V004 were 3603 and 4027, respectively, which did not differ significantly after one round (V001, 3546; V004, 2737) and two rounds (V001, 3372; V004, 3971) of stimulation. Of the unique reads identified in unstimulated PBMCs, the frequencies of shared *TRB* reads were 3.50% and 3.80% for V001 and V004, respectively, 3.19% and 5.30% for V001 and V004 after one round of stimulation, respectively, and 2.58% and 3.32% for V001 and V004 after two rounds of stimulation, respectively. Despite the low frequencies of shared unique reads as described above, shared CMV-NLV TCR CDR3b clonotypes occupied a higher rank of read numbers compared with those of the unshared CMV-NLV TCRs. In particular, the top two TCR clonotypes expressed by all unstimulated and stimulated samples from V001 and unstimulated samples from V004 were shared among all five donors (Fig. 5), although V003 and V005 were HLA-A*02-negative. Moreover, the top three TCR clonotypes in all samples of V001 and V004 represented >80% of the total reads (Fig. 5). The Wilcoxon-rank-sum test indicated that shared TCR clonotypes represented higher number of reads compared with those of unshared TCRs (Fig. 5).

## Discussion

In this study, we comprehensively investigated the TCR repertoire of functional CD8+ T cell populations at a single-cell clonotype level in circulating human blood, including naïve and memory functional T-cell subsets and the CMV pp65 NLV peptide-specific T-cell repertoire. We also evaluated the function of identified CMV NLV-specific TCRs by transducing these TCRs into T cells obtained from CMV-seronegative donors, because such knowledge is important for determining details of the mechanisms of cellular immunity in humans as well as for choosing a highly functional TCR suitable for personalized cellular immunotherapy^1, 2^. For this purpose, we employed simultaneous deep sequencing and single-cell multiplex sequencing in parallel. Although deep sequencing identifies an extensive list of TCRα or TCRβ clonotypes, it does not reveal the heterodimeric TCRαβ pairs expressed by a single T cell that recognize the MHC/peptide complex. Moreover, despite the use of high-throughput deep sequencing and bioinformatics techniques to estimate the frequencies of TCRαβ pairs^11^, the most precise and reliable method is to simultaneously determine the sequences of TCRα and TCRβ cDNAs prepared from a single T cell^3, 12^.

By combining these technologies, we identified a set of T-cell clonotypes that were frequently shared between different individuals. Interestingly, these “shared TCRs” were consistently detected in the repertoires of every functional T-cell subset (naïve, SCM, CM, EM, and EFF) and in the CMV NLV-specific T-cell repertoire. Further, functional analysis of CMV A*02/NLV-specific transduced TCRs revealed that the more dominant CMV NLV-specific TCRs had higher epitope-binding affinities and arose from the more highly shared clonotypes. Important to note is that these highly shared HLA-A*02-restricted CMV-specific TCR clonotypes were detected in HLA-A*02-negative as well as in CMV-seronegative donors, probably reflecting the fact that positive selection by “self” HLA antigens in the thymus is imperfect. Another possibility is that highly shared TCRs might be crossreactive to various HLA molecules and would be kept as T-cell repertoire during positive selection. The other possibility is that one CMV specific TCRβ might pair with different TCRα from CMV specific one, and the TCRαβ pair might have different specificity and vice versa. These results indicate that a small but substantial proportion of the T-cell repertoire shares TCR clonotypes (e.g. non-private clonotypes) irrespective of their types of HLA or previous antigen exposure. Thus, it is reasonable to speculate that dominant (or frequent) TCR clonotypes found in each individual are preferentially selected from shared, highly functional clonotypes, depending on their immunologic history.

Recently, the classical model of unidirectional T-cell differentiation, in the order of naïve, CM, EM to EFF T cells, was revised to include SCM T cells, which maintain their proliferative capacity, similar to naïve T cells^4, 5^. Gene expression profiles and immunological assays suggest that the SCM subset is generated from naïve T-cell subsets and is a precursor to CM subsets^6^. However, our comprehensive NGS analysis conducted here showed that the diversity of the SCM subset was lower compared with those of the CM and EM subsets but was similar to that of the EFF subset (Fig. 4), suggesting that the SCM populations function as a pool of a relatively lower number of unique functional T-cell clones, presumably derived from more differentiated T-cell subsets. Consistent with this hypothesis, the average frequency of unique reads of shared TCRβ chains expressed by SCM subsets of the five donors was 2.14%, which was the highest proportion among the five functional CD8+ T-cell subsets. Collectively, these observations suggest that the SCM subset may serve as a reservoir of highly functional antigen-experienced T cells.

Recent studies using NGS have also suggested that some naïve T cells show age-dependent clonal expansion in an antigen-independent fashion^13, 14^. Consequently, these studies have also seen many variations in the number of T-cell clones even in naïve subsets^14, 15^. A few previous studies also reported that expansion of naïve T cells can occur before their conversion into memory phenotype T cells^16, 17^ Although the clonotype expansion among the naïve population in this study might be more prominent when compared with these previous reports, we believe that our result is reasonable because apparently dominant clones as was observed in memory phenotype subsets were not present in naïve subsets (Fig. 4b and Supplementary Fig. 1) However, careful interpretation of this result is required because we cannot exclude the possibility of amplification bias that is inherent to PCR-based NGS analysis even when using a theoretically unbiased amplification. Also, when we conducted flow sorting, we cannot completely exclude the risk of contamination of T cells from unintended T cell subsets. To minimize the risk of contamination, we take a margin between each gate for each T cell subsets (Supplementary Fig. 3). As a result, we expect that the significant contamination of memory phenotype T cells to naïve subsets was unlikely, because CMV-specific clones that were abundant in memory subsets were absent or present at very low rank in naïve subsets (Fig. 4b and Supplementary Fig. 1). It is of note here that a new subset of memory CD8+ T cells with a phenotype indistinguishable from conventionally defined naïve T cells was recently identifed and was shown to play a role in protective immunity against persistent viral infection^18^ Expanded clonotypes in apparently naïve fractions in our study might reflect the presence of such T memory cells with a naïve-like phenotype because the frequency of these cells was reported to increase with age. Another hypothetical explanation is that HLA serotype such as A*24, that was shared with all donors in this study, might affect the shaping of expanded naïve T-cell clonotype size.

Using the highly immunogenic CMV-NLV epitope as a model antigen, we demonstrate that the epitope binding-affinities of dominant CMV NLV-specific clonotypes were higher, and the dominant clonotypes comprised a shared TCR clonotype present at relatively higher frequencies among different individuals. Our comprehensive analysis further shows that the more dominant CMV pp65-specific clonotypes have higher epitope-binding affinities and are derived from the more highly shared clonotypes. We identified several CMV NLV-specific TCRs (001-41, 001-48, and 004-71) as highly shared TCRs with high epitope-binding affinities. These results might be influenced by the fact that CMV infection is a form of lifelong chronic infection rather than an acute one. Moreover, the sequences of the CDR3 domains of these TCRs identified in this study have not been published and are not identical to published public CDR3 motifs^7, 12, 19–23^. Ethnic differences among T-cell repertoires^24^ or virus-specific immune responses (e.g. against Epstein-Barr virus^25^) may explain this difference, because most published CMV-specific TCR sequences were acquired from analyses of white populations.

In summary, the integrated bioinformatics methodology employed here is useful for expanding our knowledge of T-cell immunity. Our observations suggest that the number of functional TCR clonotypes in a given individual is relatively small, and these clonotypes are frequently shared among different individuals. These findings have encouraged our investigation of highly functional TCRs that can be used for adoptive T-cell therapy with T cells that express transgenes encoding TCRs with available sequence data. We are currently extending our analysis to include other viral or tumor-specific antigens to gain a better understanding of the origin and hierarchy of immunodominant TCRs.

## Methods

### Donor samples

This study was conducted in accordance with the principles of the Declaration of Helsinki, and all the experiments using human samples were performed according to a protocol approved by the Institutional Review Board of Hiroshima University. Peripheral blood mononuclear cells (PBMCs) were obtained from five healthy donors who provided written informed consent. All donors were screened for CMV serostatus and genotyped for HLA-A, -B, -C, -DRB1, -DQB1 and -DPB1 alleles using high-resolution Luminex methodology. PBMCs were isolated using a standard Ficoll gradient separation protocol and were stored in liquid nitrogen.

### Flow cytometric analysis and cell sorting

The expression of cell-surface molecules was determined using fluorescently-labelled monoclonal antibodies (mAbs) as follows: allophycocyanin (APC)-conjugated or fluorescein isothiocyanate (FITC)-conjugated anti-CD8, allophycocyanin-hilite7 (APC-H7)-conjugated anti-CD3, phycoerythrin-cyanine7 (PE-Cy7)-conjugated anti-CD45RO mAb, brilliant violet 510 (BV510)-conjugated anti-CD62L mAb, brilliant violet421 (BV421)-conjugated anti-CD197 mAb, APC-conjugated anti-CD95 and APC-conjucated anti-TCRαβ. These antibodies were purchased from BD Bioscience (San Jose, CA). CMV pp65-specific T cells were reacted with phycoerythrin (PE)-conjugated HLA-A*02–peptide tetramer as previously described^26^. In brief, the tetramer was made in house by one of co-author (K.K). CD8 binding site on MHC-I of the tetramer is intact. We chose an NLVPMVATV sequence of the HLA-A*02-restricted CMV pp65 peptide (NLV-peptide) as a model antigen. MHC-tetramer staining was conducted at room temperature for 15 min before cell-surface staining, which was performed at 4 °C for 30 min. The concentration of tetramer used for all experiments other than serial dilution experiments was 10 μg/ml. We checked the non-specific tetramer staining using a negative control tetramer [HLA-A2-HIV(KLTPLCVTL) tetramer-PE].

Flow cytometric analysis and cell sorting were performed using a FACSCanto II (BD Biosciences, San Jose, CA) and a FACSAria (BD Biosciences, San Jose, CA), respectively. All flow cytometry data were analysed using FlowJo software (Tree Star, Ashland, OR). We used 7-AAD to eliminate dead and damaged cells, and FSC-A/FSC-H and SSC-A/SSC-H to eliminate doublet cells. CD3+ CD8+ T cells were further subfractioned into functional subsets defined as follows^27^: naïve, CD45RO−CD62L+ CCR7+ CD95−; SCM, CD45RO−CD62L+CCR7+CD95+; CM, CD45RO+CD62L+CCR7+; EM, CD45RO+CD62L−CCR7−; EFF, CD45RO−CD62L−CCR7−. The gating strategies for cell sorting are shown in Supplementary Fig. 3.

### Cell culture

PBMCs and sorted CD8+ T cells were cultured in X-VIVO 20 (Lonza, Walkersville, MD) containing 10% AB serum, 2 mmol/l L-glutamine and 1% penicillin/streptomycin (CTL medium). B lymphoblastoid cell lines (B-LCLs) were cultured in RPMI 1640 (Sigma-Aldrich, St Louis, MO) containing 10% FBS, 2 mmol/l L-glutamine, and 1% penicillin/streptomycin. All cells were cultured in humidified incubators at 37 °C in an atmosphere containing 5% CO_2_.

Phytohemagglutinin (PHA) blasts were generated by culturing PBMCs in CTL medium containing 5 µg/ml PHA-L (Sigma-Aldrich, St Louis, MO), and the next day, IL-2 (Peprotech, Rocky Hill, NJ) was added to a final concentration of 50 U/ml. One-half of the medium was then replaced with fresh medium containing IL-2 (50 U/ml) and IL-7 (Peprotech, Rocky Hill, NJ) (20 ng/ml) twice each week. The PHA blasts were used 14 days after initiating the culture.

Jurkat cells engineered to lack TCR expression by CRISPR-Cas9 was established as follows. Briefly, following CRISPR/Cas9-mediated knock out of the endogenous TCRα-chain, CD3-negative cells were enriched by flow sorting. The sorted cells were transduced by a episomal vector containing a TCRα-chain, then CD3-positive cells (Jurkat cells with transduced α- and endogenous β-chains) were enriched by flow sorting. Endogenous TCRβ of the sorted cells were knocked out by CRISPR/Cas9, then CD3-negative cells (Jurkat cells without endogenous TCRα and TCRβ) were enriched. Then, single cell cloning of the Jurkat cells was performed using single cell sorting method by flow cytometry. Finally, a TCRβ-chain was transduced to the cloned Jurkat cells, then all of endogenous TCRα, endogenous TCRβ and transduced TCRα negative Jurkat clone was selected. TCRα negative of the clone was confirmed by transducing TCRβ and TCRβ negative of the clone was confirmed by transducing TCRα to the clone. This clone was also transduced by pMX-CD8α expression vector and was brightly stained by anti-CD8 mAb.

### *In vitro* stimulation of CMV pp65-specific T cells

CD8+ T cells were isolated from PBMCs using CD8 Microbeads (Miltenyi Biotec, Auburn, CA). CD4+ cells were depleted from the remaining cells using a CD4+ T-cell isolation kit (Miltenyi Biotec, Auburn, CA). The remaining CD4/CD8 double-negative cells were used as antigen-presenting cells (APCs). After irradiation (35 Gy), APCs were exposed to the NLV peptide (1 μM) for 2 h at room temperature and co-cultured with an equal number of CD8+ T cells in CTL medium containing IL-2 and IL-7. The synthetic NLV peptide was purchased from GenScript (Piscataway, NJ). One-half of the medium was changed twice each week.

### Semi-quantitative analysis of the TCR repertoire using high-throughput NGS

The protocol used for comprehensive TCR repertoire characterization using the unbiased gene amplification method with adaptor-ligation PCR and NGS is presented in detail in Supplementary Methods 1. Briefly, total RNA was extracted from PBMCs (5 × 10^6^), or sorted T cells and converted to cDNA with the BSL-18E primer containing poly(T)_18_ and a NotI site. Thereafter, double-strand (ds) DNA was synthesized and blunted using T4 DNA polymerase (Invitrogen). P10EA/P20EA adaptors were ligated to the 5′ end of the dsDNA and then cut with NotI. After removal of adaptors and primers, PCR was performed using *TRA* constant region-specific or *TRB* constant region-specific primers and P20EA. The second PCR was performed with constant region-specific P20EA primers using the same PCR conditions. The second-PCR products were used for high-throughput sequencing using an Illumina MiSEQ platform. After removal of sequences with low quality scores, TCR repertoire analysis was performed using bioinformatics software created by Repertoire Genesis Incorporation (Ibaraki, Japan).

### Single-cell sorting and RT-PCR

To identify and characterize CMV NLV-specific TCRαβ pairs expressed by single cells, we used a modified hTEC10 system^3, 28^ as follows: CD8/NLV tetramer double-positive cells were sorted into each well of a 96-well PCR plate. The cDNAs were synthesized and amplified using multiplex RT-PCR. The gene-specific primers used to amplify sequences encoding TCRα-chains and TCRβ-chains were designed from leader peptide sequences obtained from the IMGT database (http://www.imgt.org/). PCR reactions are described in detail in Supplementary Methods 2. TCR repertoire analysis was performed using the IMGT/V-Quest tool (http://www.imgt.org/)^29^.

### Subcloning of CMV pp65 NLV-specific *TRA* and *TRB* cDNAs

cDNAs encoding CMV pp65 NLV-specific TCRα-chains or TCRβ-chain were subcloned into the pMXs-IRES-EGFP expression vector using Gibson assembly (NEB, Ipswich, MA), which allows the joining of multiple DNA fragments in a single reaction^3^. The pMXs-IRES-EGFP plasmid was digested with BamHI and NotI, and all TCR regions were PCR-amplified using specific primer-pairs as follows: 5′-TGGAGGAGAACCCTGGACCT-3′ and 5′-GGTGAATAGGCAGACAGACTT-3′, variable region of TCRα; 5′-TGCCGGATCTAGCTAGTTAATTAAGGATCCGAATTCCTGCAGG-3′ and 5′-TTCACCCACCAGCTCAGCTC-3′, variable region of TCRβ; 5′-GAGACTCTAAATCCAGTGAC-3′ and 5′-GGGGGCGGAATTTACGTAGCGGCCGCTCAGCTGCT-3′, constant region of TCRα and 5′-TTCACCCACCAGCTCAGCTC-3′ and 5′-AGGTCCAGGGTTCTCCTCCA-3′, constant region of TCRβ. These primers were designed with synthetic overlapping ends for subcloning into a vector using Gibson assembly. To avoid mispairing and increase specific chain-pairing, the TCRα-and TCRβ-chains were joined with the porcine teschovirus-1 2A (P2A) sequence, and a Cys codon was inserted into the constant region of each TCR chain. The PCR conditions for amplifying each TCR region were as follows: 1 min at 98 °C followed by 35 cycles for 10 s each at 98 °C, 5 s at 55 °C and 1 min at 72 °C. The Gibson assembly reaction was performed according to the manufacturer’s instructions.

### Transduction of PHA blasts and TCRαβ-null Jurkat cells with sequences encoding the TCRα-chains and TCRβ-chains

We conducted retroviral transfection and nucleofection and found that their efficiencies were similar. We therefore used the latter in subsequent experiments because of its convenience. PHA blasts or TCRαβ-null Jurkat cells (1 × 10^6^) were resuspended in 20 µl of Nucleofector solution (P3 Primary Cell 4D-Nucleofector X Kit, Lonza, Walkersville, MD) containing 1 µg of TCR expression vector DNA. The cells were transduced using program EO115 of the 4D Nucleofector device. We used flow cytometry to measure GFP levels to determine transduction efficacy 6–12 h later. We used GFP-sorted cells in all subsequent functional assays.

### NLV peptide binding-affinity assay of CMV pp65 NLV-specific TCRs

To determine the binding affinities of TCRs for the NLV peptide/MHC complex, CMV pp65-NLV-specific TCR-transduced TCRαβ-null Jurkat cells were incubated with serial dilutions of the NLV tetramer. The TCR positive cells were gated on using a flow cytometer, then the mean fluorescence intensity (MFI) was determined^30, 31^. The CMV pp65-NLV-specific TCR-transduced TCRαβ-null Jurkat cells were also stained using a negative control tetramer (HLA-A2-HIV(KLTPLCVTL) tetramer-PE) to check the non-specific tetramer staining, which revealed that the NLV-tetramer that we used in this study was binding in a TCR specific manner (Supplementary Fig. 4).

### Analysis of cytokine secretion

To determine the levels of secreted cytokines, 5 × 10^5^ CMV pp65 NLV-specific TCR-transduced PHA blasts were treated with 1 × 10^5^ B-LCLs pulsed with the NLV peptide (1 μM). After 16 h incubation at 37 °C in an atmosphere containing 5% CO_2_, the supernatants were harvested and interferon (IFN)-γ production was measured using an enzyme-linked immunosorbent assay (ELISA). The results represent the mean ± standard deviation (S.D.) of triplicate experiments.

### Simpson’s Diversity Index

Simpson’s Index (D) was calculated as below (n; read number of a particular TCR, N; total read number of TCR in a sample)^32^. In this study, Simpson’s Diversity Index is calculated as 1-D. The value of this index ranges between 0 and 1. The greater the value, the greater the sample diversity. This index (1-D) is also known as Gini-Simpson Index^33^.

$${\rm{D}}=\sum {({\rm{n}}/{\rm{N}})}^{2}$$

### Statistical analysis

Difference in the IFN-γ production in ELISA between two samples were tested for significance using the paired t-test. Correlation between the MFIs of TCR-null Jurkat cells transduced with CMV NLV-specific TCRs and the estimated frequencies of clonotypes was assessed using Spearman’s rank correlation coefficient. To determine whether shared TCRs occupied the higher rank of the read number compared with unshared TCRs, we performed the nonparametric Mann–Whitney test. To assess whether the shared number and the frequency of the clonotypes would be statistically dependent, Kendall’s tau test was used. Two-sided *P* < 0.05 was considered statistically significant. All statistical analyses were performed using the STATA 12.1 software package (StataCorp, College Station, TX) or R version 3.2.3 (R Foundation, Vienna, Austria).

## Electronic supplementary material

Dataset 1
