# Supplementary material for: Highly functional T-cell receptor repertoires are abundant in stem memory T cells and highly shared among individuals
Source: Sci Rep. 2017 Jun 16;7:3663. doi: 10.1038/s41598-017-03855-x (PMC5473819; doi:10.1038/s41598-017-03855-x)
Supplement: Supplementary file 1 — Dataset 1 [file 41598_2017_3855_MOESM1_ESM.doc]

**Supplementary Information**

**for**

**Highly functional T-cell receptor repertoires are abundant in stem memory T cells and highly shared among individuals**

Takahiko Miyama, Takakazu Kawase, Kazutaka Kitaura, Ren Chishaki, Masashi Shibata, Kumi Oshima, Hiroshi Hamana, Hiroyuki Kishi, Atsushi Muraguchi, Kiyotaka Kuzushima, Hiroh Saji, Tadasu Shini, Ryuji Suzuki & Tatsuo Ichinohe

**Supplementary Figure 1. Association between the read number of TCRβ clonotypes and the number of individuals who share the clonotypes in the naïve, SCM, CM, EM and EFF subsets of CD8+ T cells identified using NGS analysis.** Random sampling of 35,000 reads was performed in each T cell subset and each donor. Each circle or dot in the plots shows one unique read of TCRβ. TCRβ clonotypes are ordered according to their frequencies in descending order on the x-axis. The y-axis depicts the read number of each clonotype. Shared non-CMV NLV-specific TCRs (○) and unshared non CMV NLV-specific TCRs (●) are shown as black circles and blue dots, respectively. Shared CMV NLV-specific TCRs (○) and unshared CMV NLV-specific TCRs (●) are shown as red circles and dots, respectively. Orange thick circle (**○**) represents one of nine dominant TCRs in V001 or V004 (TCR ID; 001-17, 001-48, 001-41, 004-66, 004-22, 004-63, 004-30, 004-28 or 004-71) (Figure 2a). The sizes of the circles are directory proportional to numbers shared among the five donors. The largest circles indicate the shared clonotypes by the five donors and the dots indicat the unshared clonotypes. Wilcoxon rank-sum test was performed. Additionally, Kendall’s tau test was used to assess the association between the sharing count and the frequency of the shared clonotypes. The two-sided *P* values of all tests were <0.05, which indicates higher frequencies of shared TCRs compared with unshared TCRs and a significant correlation between the total read number and the sharing count of the shared TCRs, even in the naïve subset. The results of this analysis for donors V003 and V004 are shown in Figure 4B.

**Supplementary Figure 2. T cell repertoire plots of functional CD8+ T-cell subsets for donors V001, V002 and V005.** T cell repertoire plots of functional CD8+ T-cell subsets are the same as shown in Fig. 4c, revealing the lower diversity of the SCM T-cell subset compared with the CM and EM T cell subsets. The results of this analysis were similar for donors V003 and V004 (Fig. 4d).

**Supplementary Figure 3.** **Sorting of functional CD8+ T-cell subsets of PMBCs.** Dot plots shows the gating strategy used to separate functional CD8+ T-cell subsets. Dead and damaged cells were eliminated using 7-AAD. Doublet cells were eliminated using FSC-A/FSC-H and SSC-A/SSC-H. Functional T-cell subsets were defined as follows: naïve, CD3+CD8+CD45RO-CD62L+CCR7+ CD95–; Stem cell memory (SCM), CD3+CD8+CD45RO-CD62L+CCR7+ CD95+; Central memory (CM), CD3+CD8+CD45RO+CD62L+CCR7+; Effector memory (EM), CD3+CD8+CD45RO+CD62L-CCR7- and Terminal effector (EFF), CD3+CD8+CD45RO-CD62L-CCR7-.

**Supplementary Figure 4. Staining using a negative control tetramer of the CMV pp65-NLV-specific TCR-transduced TCRαβ-null Jurkat cells.** TCRαβ-null Jurkat cells transduced with the CMV pp65-NLV-specific TCR (Fig. 3a) were stained using a negative control tetramer [HLA-A*02-HIV (KLTPLCVTL) tetramer-PE] to check the non-specific tetramer staining.

**Supplementary Table 1. Comparison between Simpson's Index calculated using all reads and random sampling data**. SCM, stem cell memory; CM, central memory; EM, effector memory; EFF, terminal effector; SD, standard deviation.

**Supplementary Table 2. Frequencies of unique and total reads of shared and unshared TCRβ-chains of naïve, SCM, CM, EM, and EFF CD8+ T-cell subsets obtained from peripheral blood.** SCM, stem cell memory; CM, central memory; EM, effector memory; EFF, terminal effector; NUR, number of unique reads; NTR, number of total reads.

**Supplementary Table 3. Primers for next-generation sequencing of rearranged T-cell receptor gene segments**

| **Primer** | **Sequence** | **MID Tag** |
| --- | --- | --- |
| BSL-18E | AAAGCGGCCGCATGCTTTTTTTTTTTTTTTTTTVN |  |
| P20EA | TAATACGACTCCGAATTCCC |  |
| P10EA | GGGAATTCGG |  |
| CA1 | TGTTGAAGGCGTTTGCACATGCA |  |
| CA2 | GTGCATAGACCTCATGTCTAGCA |  |
| CB1 | GAACTGGACTTGACAGCGGAACT |  |
| CB2 | AGGCAGTATCTGGAGTCATTGAG |  |
| HuVaF-01~10 | **CCATCTCATCCCTGCGTGTCTCCGAC**TCAG-{MID}-ATAGGCAGACAGACTTGTCACTG | MID1~MID11 |
| HuVbF-01~10 | **CCATCTCATCCCTGCGTGTCTCCGAC**TCAG-{MID}-ACACCAGTGTGGCCTTTTGGGTG | MID15~MID24 |
| B-P20EA | ***CCTATCCCCTGTGTGCCTTGGCAGTC***TAATACGACTCCGAATTCCC |  |

V: A/C/G, N: A/C/G/T, Adaptor A and B sequences were indicated in bold and bold italics, respectively. A key sequence (TCAG) is underlined. The MID Tag sequences used for identification of the sample source were as follows: MID1, ACGAGTGCGT; MID2, ACGCTCGACA; MID3, AGACGCACTC; MID4, AGCACTGTAG; MID5, ATCAGACACG; MID6, ATATCGCGAG; MID7, CGTGTCTCTA; MID8, CTCGCGTGTC; MID10, TCTCTATGCG; MID11, TGATACGTCT; MID15, TACGACGTA; MID16, TCACGTACTA; MID17, CGTCTAGTAC; MID18, TCTACGTAGC; MID19, TGTACTACTC; MID20, ACGACTACAG; MID21, CGTAGACTAG; MID22, TACGAGTATG; MID23, TACTCTCGTG; MID24, TAGAGACGAG.

**Supplementary Methods 1.**

**Unbiased amplification of TCR genes**

Total RNA was extracted from PBMCs or sorted T cells using the RNeasy Lipid Tissue Mini Kit (Qiagen, Hilden, Germany) according to the manufacturer’s instructions. RNA amounts and purity were measured using an Agilent 2200 TapeStation (Agilent Technologies, Palo Alto, CA). One microgram of total RNA was converted to cDNA using Superscript III reverse transcriptase (Invitrogen, Carlsbad, CA). The BSL-18E primer containing poly(T)_18_ and a NotI site was used for cDNA synthesis. After cDNA synthesis, double-strand (ds)-cDNA was synthesized using *Escherichia coli* DNA polymerase I (Invitrogen), *E. coli* DNA Ligase (Invitrogen) and RNase H (Invitrogen). The ends of the ds-cDNAs were blunted using T4 DNA polymerase (Invitrogen). The P10EA/P20EA adaptor was ligated to the 5´ end of the ds-cDNA and then cleaved with NotI. After removal of the adaptor and primer with a MinElute Reaction Cleanup kit (Qiagen), PCR was performed using primers, either the TCRα-chain constant region-specific (CA1) or TCRβ-chain constant region-specific primers (CB1) and P20EA (Supplementary Table 3). PCR conditions were as follows: 95 °C (30 s), 55 °C (30 s), and 72 °C (1 min) for 20 cycles. The second PCR was performed with either CA2 or CB2 and P20EA primers using the same PCR conditions.

**Amplicon sequencing using a Roche 454 Sequencing System**

Amplicons for NGS were prepared from the second-round PCR products using the P20EA primer and the fusion Tag primer (Supplementary Table 3). The fusion Tag primers comprised an A-adaptor sequence (CCATCTCATCCCTGCGTGTCTCCGAC), 4-base sequence key (TCAG), and multiple identifier (MID) Tag sequence (10 nucleotides). TCR constant region-specific sequences were designed according to the manufacturer’s instructions. After PCR amplification, amplicons were evaluated using agarose gel electrophoresis. Incomplete fragments or primers were removed using Agencourt AMPure XP (Beckman Coulter, Brea, CA) according to the manufacturer’s instructions. The amounts of purified amplicons were quantified using a Quant-iT PicoGreen dsDNA Assay Kit (Life Technologies, Carlsbad, CA). Each amplicon obtained with a different fusion Tag primer from 10 samples was mixed at equimolar concentrations. Emulsion PCR (emPCR) was performed using the amplicon mixtures with a GS Junior Titanium emPCR Lib-L kit (Roche 454 Life Sciences, Branford, CT) according to the manufacturer’s instructions.

**Assignment of *TRV* and *TRJ* segments**

All sequence reads were classified according to their MID Tag sequences. Artificially added sequences (Tag, adaptor, and key) and sequences with low quality scores were removed from both termini of sequence reads using software supplied with the 454 Sequencing System. The remaining sequences were used for assignment of *TRAV* and *TRAJ* of TCRα sequences, and *TRBV* and *TRBJ* for TCRβ sequences. Assignment of sequences was performed by determining those with the highest percentage identities in a dataset of reference sequences for 54 *TRAV*, 61 *TRAJ*, 65 *TRBV* and 14 *TRBJ* genes including pseudogenes and open reading frame (ORF) reference sequences available from the international ImMunoGeneTics Information System (IMGT) database (http://www.imgt.org). Data processing, assignment, and data aggregation were automatically performed using repertoire analysis software (Repertoire Genesis, RG) originally developed by Repertoire Genesis Incorporation (Osaka, Japan). RG first assigns *TRV* and *TRJ* alleles to queries using BLASTN and the IMGT TCR data set. Identity between query and reference sequences is calculated in this step. Parameters that increase sensitivity and accuracy (E-value threshold, minimum kernel, high-scoring segment pair (HSP) score) were optimized for the respective repertoire analysis. RG then estimates the CDR3 regions of queries by considering their reading frames, which are translated. RG then calculates the distribution of TRV-CDR3-TRJ patterns and generates graphics such as TRV-TRJ usage histograms and CDR3 length-distribution charts. These steps are all performed automatically after the researcher enters the query.

**Data analyses**

Translated nucleotide sequences of the *CDR3* regions ranged from the conserved Cys104, according to the IMGT nomenclature, to the conserved Phe118, or Gly119. A unique sequence read (USR) was defined as 0% identity to *TRV*, *TRJ* and the deduced amino acid sequence of the CDR3 domains of the other sequence reads. RG software automatically counted the copy numbers of identical USRs in each sample and then ranked them in order of copy number. Percentage frequencies of sequence reads of *TRAV*, *TRAJ*, *TRBV* and *TRBJ* genes were calculated.

**Supplementary Methods 2.**

**RT-PCR analysis of *TCRA* and *TCRB* pairs**

CD8/NLV tetramer double-positive cells were sorted into each well of a 96-well PCR plate. cDNAs were directly synthesized and amplified from each cell using multiplex RT-PCR. The gene-specific primers for TCR**α** and TCRβ were designed from leader peptide sequences obtained from the IMGT database (http://www.imgt.org/). RT-PCR was performed in a reaction mixture containing 0.1 µl of 40 U/µl RNase Inhibitor (NEB, Ipswich, MA), 0.1 µl of 200 U/µl PrimeScript II RTase (TaKaRa, Otsu, Japan), 0.4 µl primer mixture, 0.025 µl of 2.5 U/µl PrimeStar HS DNA Polymerase (TaKaRa), 0.4 µl of 2.5 mM dNTP and 2.5 µl of 5×PrimeStar GC buffer (TaKaRa). DEPC-treated H_2_O was added to a final volume of 5 µl. The RT reaction was performed at 45 ºC for 40 min followed by PCR as follows: 1 min at 98 ºC, followed by 30 cycles for 10 s at 98 ºC, 5 s at 55 ºC and 1 min at 72 ºC. The PCR reactions were diluted 10-fold with water and used as template DNAs for the subsequent nested PCRs. The nested PCR for amplified *TCRA* and *TCRB* were performed in different 96-well PCR plates. The reaction mixture contained 2 µl of DNA template from the first PCR reaction, 0.4 µl of 10 µM of the respective specific primer set (A-AD and A-RV2 primers for TCRα, B-AD and B1-RV2 primers, B2-RV2 primer for TCRβ, 0.1 µl of 2.5 U/µl PrimeSTAR HS DNA Polymerase, 1.6 µl of 2.5 mM dNTP, 10 µl of 5 × PrimeSTAR GC Buffer, 0.1 µl of 2.5 U/µl and H_2_O added to a final volume of 20 µl. The PCR cycles were as follows: 1 min at 98 ºC, followed by 35 cycles for 10 s at 98 ºC, 5 sec at 55 ºC and 1 min at 72 ºC. The *TCRA* and *TCRB* PCR products were analyzed using Sanger sequencing.
